# Sign- and goal-tracking score does not correlate with addiction-like behavior following prolonged cocaine self-administration

**DOI:** 10.1007/s00213-021-05858-z

**Published:** 2021-05-05

**Authors:** Veronika Pohořalá, Thomas Enkel, Dusan Bartsch, Rainer Spanagel, Rick E. Bernardi

**Affiliations:** 1 Institute of Psychopharmacology, Central Institute of Mental Health, Medical Faculty Mannheim, University of Heidelberg, J5, 68159 Mannheim, Germany; 2 Department of Molecular Biology, Central Institute of Mental Health, Medical Faculty Mannheim, University of Heidelberg, Mannheim, Germany

**Keywords:** Pavlovian conditioned approach, Rats, Sign trackers, Goal trackers, 3-CRIT, Cocaine, Self-administration, Addiction

## Abstract

**Rationale:**

In classical conditioning, sign-tracking reflects behavior directed toward a conditioned stimulus (CS) in expectation of a reward (unconditioned stimulus, US); in contrast, goal-tracking describes behavior directed toward the location of delivery of a US. As cues previously paired with drugs of abuse promote drug-seeking and drug-taking behavior in both animals and humans and thus contribute to the severity of substance abuse, sign-tracking may represent a maladaptive cue-focused behavior that may increase addiction vulnerability as compared to goal-tracking. Recent studies do, in fact, support this possibility. Previous work in this area has focused primarily on paradigms using relatively limited exposure to drug rather than extended drug intake.

**Objectives:**

Here, we used the DSM-IV–based 3-criteria (3-CRIT) model and examined whether a relationship exists between sign- or goal-tracking phenotypes and the prevalence of criteria associated with addiction-like behavior following extended cocaine self-administration as measured in this model.

**Methods:**

Forty-six male Sprague Dawley rats underwent a Pavlovian conditioned approach (PCA) procedure and were characterized along a continuum as goal-trackers (GTs), intermediates (INTs), or sign-trackers (STs). The animals were subsequently trained to intravenous self-administer cocaine during 45 self-administration (SA) sessions and characterized for the 3 criteria outlined in the model: persistence of drug-seeking, motivation for cocaine-taking, and resistance to punishment.

**Results:**

We performed correlational analyses on the traits measured, finding no relationships between PCA score and addiction-like characteristics measured using the 3-CRIT model of addiction. However, STs showed significantly greater resistance to punishment than GTs.

**Conclusions:**

Phenotyping along a continuum of PCA scores may not be a valid predictor for identifying vulnerability to the addiction-like behaviors examined using the 3-CRIT model. However, PCA phenotype may predict a single feature of the 3-CRIT model, resistance to punishment, among those rats classified as either STs or GTs.

## Introduction

Substance use disorder (SUD) is a complex and chronic condition characterized by an individual’s loss of control over drug-taking and drug-seeking, together with prolonged drug intake despite negative consequences and a high susceptibility to relapse. Relapse, which occurs even after long periods of abstinence, is a major obstacle in the treatment of SUD (Hunt et al. 1971; Leshner 1997; O’Brien 2005), with more than 80% of dependent individuals relapsing to drugs of abuse (Brandon et al. 2007). Drug-associated stimuli are a major contributor to relapse, highlighted by numerous demonstrations that contextual and discrete stimuli previously associated with drug intake (environment, persons, objects, etc.) can promote drug-seeking and drug-craving in humans (e.g., Mayo et al. 2013; O’Brien 2005). Because an individual’s response in the presence of drug-associated stimuli or environments undoubtedly varies as a function of that individual’s unique experiences in the context of drug-seeking and drug-taking, it is important to develop behavioral assays that help to gauge an individual’s susceptibility to relapse in the presence of drug cues in order to better develop treatment strategies for dependence.

Sign-tracking and goal-tracking procedures in humans have been developed in an attempt to categorize humans into sign-trackers (STs) or goal-trackers (GTs) based on consistent attentional approach behavior—typically eye-tracking measurements—toward a conditioned stimulus (CS) or an unconditioned stimulus (US), respectively (Garofalo and di Pellegrino 2015; Le Pelley et al. 2015; Schad et al. 2020). An attentional bias toward reward-paired stimuli, rather than the reward or goal itself, is thought to be due to an attribution of incentive salience to such stimuli (Anderson et al. 2013; Le Pelley et al. 2016). For example, Garofalo and di Pellegrino (2015) reported a higher tendency of STs to respond to task-irrelevant CS when the US was not available. Furthermore, Schad et al. (2020) demonstrated that the CS in their experiment induced more incentive salience and elicited more attentional approach behavior in STs. Furthermore, the authors used neuroimaging and computational modeling to demonstrate that STs rely on dopaminergic model-free learning in which learning occurs via reward prediction error (see Schultz et al. 1997), while GTs rely on non-dopaminergic model-based learning in which learning occurs via state prediction error (see Glascher et al. 2010). These findings suggest two dissociable learning processes within any given individual, the balance of which likely determines how an individual will respond to a new experience and will likely respond to future experiences (Schad et al. 2020).

Because sign- and goal-tracking procedures in humans have not yet progressed to reliably evaluate differences in behaviors associated with drug dependence, these procedures in animals—despite potential behavioral, neurobiological, and cognitive differences relative to those in humans (reviewed in Colaizzi et al. 2020)—remain vital in measuring the extent to which CS govern instrumental behaviors. In rodents, the reaction of animals to a given CS is measured using the Pavlovian conditioned approach (PCA), with some animals approaching and focusing on the CS itself, by gnawing, biting, and licking the CS, oftentimes a lever (STs), and others approaching the location of the US (e.g., food; GTs), even as delivery of the reward is independent of the behavior of the animal. While both STs and GTs acquire knowledge of the CS-US association, as measured by both groups learning a conditioned orienting response (Robinson et al. 2014; Yager and Robinson 2013), STs appear to respond to a greater extent to the incentive motivational properties of the CS, while GTs focus more on its predictive properties (Bolles 1972; Enkel et al. 2019; Yager and Robinson 2010). These findings have generated the idea of a possible link between a ST phenotype and addiction (Flagel et al. 2009; Saunders and Robinson 2013).

In terms of drugs of abuse, several animal studies have demonstrated that STs show increased responding in the presence of drug and drug-related cues. For example, a study using the conditioned cue preference procedure (a modification of conditioned place preference) demonstrated that STs prefer the cocaine-associated floor more than GTs, likely due to stronger conditioned reinforcing properties of the cocaine-associated floor in STs (Meyer et al. 2012). In addition, another study (Hilz et al. 2019) using CPP with a different psychostimulant, amphetamine, in the context of conditioned orienting as a form of sign-tracking behavior, showed that STs (in the study referred to as “orienters”) preferred the amphetamine-associated surroundings to a greater extent and this preference was more resistant to extinction. Furthermore, it has been demonstrated that STs work harder than GTs for cocaine under a progressive ratio (PR) schedule (Saunders and Robinson 2011) and show more robust cue- and drug-induced reinstatement of drug-seeking (Saunders and Robinson 2010, 2011; Yager and Robinson 2013). Furthermore, STs also choose cocaine over food significantly more than GTs when given a choice between cocaine infusion and food; although only a small subset of rats (19%) chose cocaine over food, all of them were STs (Tunstall and Kearns 2015). In contrast, Vanhille et al. (2015) reported a significant preference for cocaine over saccharin in rats but found no difference in preference between STs and GTs. Whereas most of these studies applied schedules with relatively limited exposure to drugs of abuse (varying from 5 to ~ 20 SA sessions), Kawa et al. (2016) conducted a study using a prolonged intermittent access procedure (36 SA sessions over ~ 70 days). The authors reported that after limited drug experience (14–19 SA sessions), STs were considerably more motivated to self-administer cocaine than GTs, whereas after a prolonged drug-taking period, this significant difference was no longer present. Thus, the degree of access may be a determinant of addiction-like vulnerability in animal models based on GT and ST phenotypes (Colaizzi et al. 2020).

Another protocol used for modeling addiction preclinically is the 3-criteria (CRIT) model of drug (cocaine) self-administration (SA). This model is based on addiction criteria described for human dependence in the DSM-IV and modified for rats to measure the following: the persistence of drug-seeking, motivation for drug-seeking, and drug-taking despite adverse consequences. The outcome of the 3-CRIT model is a distribution of animals ranging from those showing no addicted-like behavior (0 crit) to those showing addicted-like behavior on all three criteria (3 crit) following extended cocaine SA (Deroche-Gamonet et al. 2004). Results of the 3-CRIT model mimic the distribution of substance-dependent individuals in the human population—only a small percentage (15–17%) of drug users actually develop a chronic condition that can be characterized as addiction (Anthony et al. 1994; Lopez-Quintero et al. 2011). Therefore, the 3-CRIT model is a multi-symptomatic tool used to identify subpopulations of animals prone to (3 crit) addiction-like behavior and resilient toward (0 crit) addiction-like behavior.

The present study was undertaken to determine whether STs versus GTs show differential vulnerability to developing addicted-like criteria using the 3-CRIT model in rats. We first determined the PCA phenotype (GTs, intermediates (INTs), and STs) using a PCA paradigm measuring the propensity to approach a cue (lever) or reward (food). Animals were then subjected to extended cocaine SA, following which we measured the persistence of drug-seeking (measured as nosepokes when cocaine was unavailable), motivation for cocaine-taking (measured as breakpoint using a PR schedule), and drug-taking despite adverse consequences (measured by resistance to footshock-induced punishment). We then performed correlation analyses to identify potential statistical relationships between PCA behaviors and addiction-like behavioral characteristics.

## Materials and methods

### Subjects

All experiments were carried out at the Central Institute of Mental Health in Mannheim (Germany). Experimental procedures were conducted according to the NIH ethical guidelines for the care and use of laboratory animals, in compliance with the German Animal Welfare Act, and approved by the local animal care committee (Regierungspräsidium Karlsruhe, Germany).

Subjects were 46 male Sprague Dawley rats (Charles River, Germany), 6 weeks old at the time of arrival at the CIMH animal housing facility. The animals were housed on a reversed light cycle (lights on: 7 p.m.–7 a.m.) in 3 UniProtect air-flow cabinets (Bioscape, Germany), located in a temperature (22 °C ± 1 °C) and humidity (40% ± 5%)–controlled room. From their arrival to the end of Pavlovian conditioning training, rats were housed in groups of four. Following catheterization surgery until the end of the experiment, rats were housed individually. During training and tests, subjects received 20 g/day of standard chow food, with water provided ad libitum. A total of 45 animals finished the experiment; one animal died during 3-CRIT training. All experiments were performed during the dark phase.

### Drugs

Cocaine HCl (Sigma-Aldrich, Germany) was dissolved in sterile 0.9% NaCl for intravenous (i.v.) administration of 0.8 mg/kg/36 µl infusion.

### Pavlovian conditioned approach

The Pavlovian conditioned approach took place in conditioning chambers (21 cm × 24 cm × 29 cm; Med Associates, St. Albans, VT, USA) that contained a liquid dispenser, a pellet dispenser, a receptacle with head entry detectors, and two retractable levers (on each side of the receptacle) which required a downward force of ~ 12–15 g to record a press. All procedures were controlled by a PC-running custom-made MedStat notation code (MedPC IV, Med Associates, St. Albans, VT, USA). The general procedure followed earlier descriptions (Saunders and Robinson 2012) with minor adaptations (Enkel et al. 2019; Scülfort et al. 2016). Briefly, PCA assessment started with 20 free deliveries of the US (80 µl of a 20% sweetened condensed milk solution) on two successive days. This was followed by 7 days of acquisition; every session consisted of 20 trials: the lever (CS) was presented for 8 s, and after its retraction, the liquid dispenser provided the US. Trials were separated by intertrial intervals (ITIs; 30–115 s); however, trial onset was postponed by 8 s if a head entry occurred immediately prior to trial start. This avoided a confounding recording bias toward GT behavior due to non–CS-triggered ITI activity. During a given trial, a conditioned response (CR) was scored if any form of responding (lever deflection or food cup entry) occurred within the 8 s of CS presentation. The pattern of responding was quantified using a PCA score, consisting of the mean of three measures: the probability of lever deflection or food cup entry, the response bias for lever/food cup responses, and the latency to make lever/food cup responses (Saunders and Robinson 2012). Rats were then grouped into GTs (− 0.5 to − 1.0), INTs (− 0.5 to 0.5), and STs (+ 0.5 to + 1.0).

### Surgeries

Rats were implanted under isoflurane anesthesia with a catheter composed of a Micro-Renathane® tube (internal diameter: 0.58 mm; external diameter: 0.94 mm) and a back-mount (Plastic One Inc., USA; 313-000BM, 22 gauge). The proximal end reached the right atrium through the right jugular vein; the back-mount passed under the skin and protruded from the mid-scapular region. Rats were given ~ 5 days to recover from the surgery before the beginning of cocaine SA training. The catheter was treated every day until the end of the experiment (before and after the session) with a solution containing heparin (100 IU/ml) and enrofloxacin (Baytril®; 5 mg/kg).

### Self-administration apparatus

All 3-CRIT training and testing sessions were performed in 24 boxes for SA (40 cm × 30 cm × 52 cm; Imetronic, France) located in cabinets with fans that ensured air exchange and masked external sounds. Two nosepoke holes were located on opposite walls of the chambers, 5 cm above the grid floor. When rats poked their snout in the holes, breaking an infrared beam, an instrumental response was recorded. One hole was associated with cocaine delivery and designated as the active hole, while the other was designated as the inactive hole and served as a control. A white house light located at the top allowed the illumination of the entire chamber; a white cue light was located 9.5 cm above the active hole, a green cue light was 10 cm to the right of the white one, and a blue cue light was on the left side of the opposite wall 33 cm above the grid floor. PC Windows–compatible software (Imetronic, France) controlled all experiments.

### 3-CRIT model of addiction

All procedures used during 3-CRIT training were adapted from previously published work (Belin et al. 2009; Cannella et al. 2017, 2013; Deroche-Gamonet et al. 2004; Kasanetz et al. 2013). Animals were subjected to 45 cocaine SA sessions. Each session was 2.5 h long and included three drug periods (40 min each) alternating with two non-drug periods (15 min each). The drug period was signaled by the blue cue light, whereas non-drug periods were signaled by the illumination of the entire chamber by the house light. The required amount of nose-poking in the active hole during drug periods resulted in the illumination of the white cue light (4 s in total) that was followed 1 s later by the activation of the infusion pump (36 µl/infusion over 2 s, containing 0.8 mg/kg of cocaine). Cocaine infusion was followed by a 40-s time-out (TO) period. After 10 sessions of initial training under a fixed ratio schedule 3 (FR3), the program was switched to FR5 for the remainder of the experiment. During each cocaine self-administration session, except those during which motivation for cocaine-taking and resistance to punishment were tested, a maximum of 35 infusions was allowed. If an animal reached 35 infusions, the session ended. Nose-poking in the inactive hole was recorded but had no programmed consequences. During the non-drug periods, nose-poking in both holes was recorded but had no programmed consequences. When rats achieved 43 cocaine self-administration sessions, two criteria for addiction behavior were evaluated: persistence of drug-seeking and motivation for cocaine-taking and, following 2 days of additional cocaine SA sessions, resistance to punishment.

### Persistence of drug-seeking

This addiction criterion was evaluated daily during SA training by measuring the number of nosepokes in the active hole during the non-drug periods during which cocaine was unsignaled and unavailable. The last three SA training sessions prior to the motivation test (days 41–43) were averaged for each animal to determine the persistence of drug-seeking.

### Motivation for cocaine-taking

This criterion was assessed on session no. 44 using a PR schedule. The PR session used identical features as drug periods above, but with the ratio of responses to achieve cocaine increasing with every infusion according to the following nosepoke progression: 10, 20, 30, 45, 65, 85, 115, 145, 185, 225, 275, 325, 385, 445, 515, 585, 665, 745, 835, 925, 1025, 1125, 1235, 1345, 1465, 1585, 1715, 1845, 1985, 2125, 2275, 2425, 2585, 2745, 2915, 3085, 3265, 3445, 3635, 3825, 4025, and 4225. The last ratio completed is referred to as the breakpoint (BP) and used to score the criterion. The session ceased after 6 h or a 60-min limited hold (i.e., 1 h passed without completion of the next response ratio).

### Resistance to punishment

Following PR testing, rats were subjected to 2 days of normal SA training (days 45–46) to ensure normal responding and cocaine intake. The resistance to punishment criterion was assessed on session no. 47 by pairing cocaine-seeking and cocaine-taking with electric footshocks. The session lasted 40 min and was a modified version of the standard drug period. As usual, rats were required to achieve an FR5 ratio of responses to receive a cocaine injection. However, here, a single nosepoke led to the illumination of the green cue light, signaling the presence of footshock. When 4 nosepokes were achieved, rats received an electric footshock (0.2 mA, 1 s). Completion of the FR5 ratio then resulted in simultaneous delivery of the cocaine infusion and exposure to a 2nd footshock (0.2 mA, 1 s). From illumination of the green light, rats had 1 min to complete the 4th nosepoke and then another minute to complete the FR5 ratio; if these requirements were not met, the green cue light was extinguished and the sequence reinitiated. The blue and white cue light schedules functioned as in the standard drug period. This criterion was expressed as the percentage of cocaine infusions earned relative to the number of infusions earned during the 1st 40-min drug period on day 46.

### Characterization of addiction-like behavior

A subject was considered positive for one criterion if its score was above the 60th percentile of the population distribution, and negative if its score was below the 60th percentile (Cannella et al. 2017, 2013). Thus, depending on the number of positive criteria met, a subject was assigned to one of the following groups: 0 crit, 1 crit, 2 crit, and 3 crit. Rats negative for all the criteria (0 crit) were characterized as non-addict-like, whereas rats positive for all the criteria (3 crit) were characterized as addict-like.

### Addiction score

An addiction score was also calculated for each animal as the sum of the normalized scores of the three addiction-like criteria. To calculate the normalized score of a criterion, the average across the whole population of that criterion was subtracted from the subject score, and the result of this subtraction was divided by the standard deviation of the whole population. Thus, each subject had three normalized scores, one for each addiction criterion (motivation, persistence, resistance to punishment). In addition to the categorical classification (number of positive criteria met), this score is a dimensional index of cocaine use severity that has been shown to be linearly related to the number of criteria met, supporting the hypothesis that the model identifies a pathologic-like continuum, from controlled to compulsive-like drug use (Belin et al. 2009, 2008; Rikoon et al. 2006).

### Data analysis

Group scores for PCA (GTs, INTs, and STs) were analyzed using repeated measures ANOVA (repeated measure: day), followed by one-way ANOVA to detect between-group differences. Self-administration data were analyzed using repeated measures ANOVA (repeated measures: day and nosepoke [active, inactive]). The 3-CRIT model (motivation for cocaine-taking, persistence of cocaine-seeking, resistance to punishment, as well as addiction score) was analyzed using descriptive statistics (mean, standard error, and population distribution), with independent-samples *t* tests to indicate differences between 0 and 3 crit scores. Correlations between PCA scores and addiction criteria were performed using Pearson’s correlation coefficient (*r*). Comparisons of GTs and STs on addiction-like criteria were analyzed using independent-samples *t* tests. Significance was set at *p* = 0.05.

## Results

### PCA

**Fig. 1:**
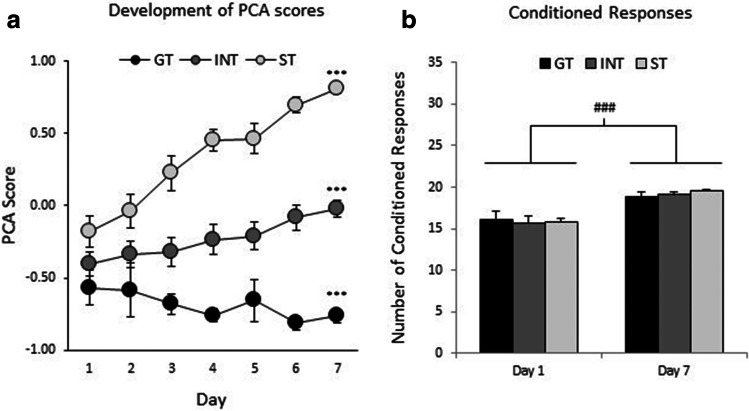
Development of PCA score and conditioned responses. **a** Rats characterized according to their PCA scores on day 7 as GT (*n* = 8), INT (*n* = 20), and ST (*n* = 17) plotted as the development of these phenotypes. Rats showed no difference in PCA scores on day 1, but a significant difference on day 7. Data represent the mean (± SEM) PCA score across 7 days of PCA sessions. **b** The number of conditioned responses did not differ among GT, INT, and ST groups on day 1 or day 7 of PCA training; however, the number of conditioned responses increased significantly from day 1 to day 7. Data represent the mean (± SEM) number of conditioned responses on days 1 and 7. ***p < 0.0005, versus all other groups; ^###^*p* < 0.0005

Following 7 days of PCA, distinct patterns of approach behaviors to the lever, the food cup, or some intermediate combination had developed. Animals were then divided into 3 groups according to their PCA scores: rats with PCA scores less than − 0.5 were categorized as GTs (*n* = 8), rats with PCA scores between − 0.5 and 0.5 were categorized as INTs (*n* = 20), and rats with PCA scores > 0.5 were categorized as STs (*n* = 17). Mean (± SEM) PCA scores for GTs, INTs, and STs on day 7 were − 0.76 ± 0.05, − 0.02 ± 0.05, and 0.81 ± 0.03, respectively. Figure 1a shows the development of mean (± SEM) PCA scores over 7 days for groups GT, INT, and ST. A two-way ANOVA of PCA scores (group × day) revealed significant main effects of group (*F*(2,42) = 51.1, *p* < 0.0005) and day (*F*(3.7,155.3) = 8.1, *p* < 0.0005) and a significant group × day interaction (*F*(7.4,155.3) = 6.5, *p* < 0.0005), indicating a difference in the development of GT, INT, and ST behaviors, and a difference between the groups. One-way ANOVA of data on days 1 and 7 confirmed that PCA scores among the groups did not differ significantly on day 1 (*F*(2,42) = 3.1, *p* = 0.06) but differed on day 7 (*F*(2,42) = 202.6, *p* < 0.0005; Bonferroni post hoc: GT vs. INT, *p* < 0.0005; GT vs. ST, *p* < 0.0005; INT vs. ST, *p* < 0.0005). The mean number of CR was similar in all three groups on both day 1 and day 7, indicating similar levels of learning and performance (Fig. 1b). A two-way ANOVA of CR (group × day) revealed a significant main effect of day (*F*(1,42) = 46.2, *p* < 0.0005), but no other significant effects (group: *F*(2,42) = 0.2, *p* = 0.85; group × day: *F*(2,42) = 0.3, *p* = 0.74). These findings indicate similar performance in all groups at the beginning and end of training, as well as an increase in the learning of the task, demonstrated by the significant increase in the number of CR from day 1 to day 7. On day 7, the mean number of lever approaches and food cup head entries, respectively, were 6.1 ± 2.4 and 149.1 ± 21.7 for GT rats, 35.8 ± 3.3 and 90.0 ± 13.5 for INT rats, and 95.8 ± 7.3 and 4.6 ± 1.5 for ST rats, respectively.

### Self-administration

**Fig. 2:**
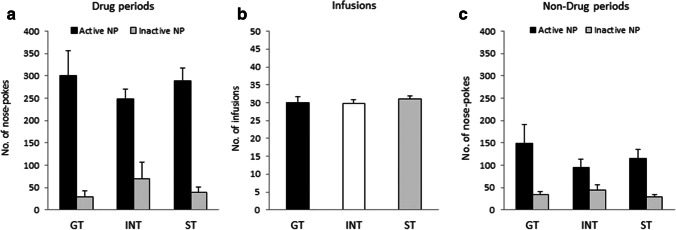
Cocaine self-administration in GTs, INTs, and STs across all FR5 sessions. There were no differences in the number of **a** nosepokes during drug periods, **b** reinforcers achieved, or **c** nosepokes during non-drug periods between GTs, INTs, and STs across sessions. Data represent the mean (± SEM) number of active and inactive nosepoke reinforcers achieved for groups GT, INT, and ST

GT, INT, and ST rats did not differ in SA behavior during drug periods, cocaine infusions received, or SA behavior during non-drug periods. Figure 2a shows the mean (± SEM) number of active and inactive nosepokes during drug periods across all FR5 SA sessions for GT, INT, and ST groups. A three-way ANOVA (PCA group × day × nosepoke) revealed a significant effect of nosepoke (*F*(1,42) = 80.7, *p* < 0.0005), but no other significant effects (group: *F*(2,42) = 0.02, *p* = 0.98; day: *F*(4.4,185.1) = 1.2, *p* = 0.30; group × day: *F*(8.8,185.1) = 0.9, *p* = 0.49; group × nosepoke: *F*(2,42) = 1.4, *p* = 0.27; day × nosepoke: *F*(4.3,178.8) = 0.6, *p* = 0.65; group × day × nosepoke: *F*(8.5,178.8) = 1.0, *p* = 0.44), indicating a distinction between the active and inactive nosepokes, but no other differences between the groups across sessions. Figure 2b shows the mean (± SEM) number of cocaine infusions across all FR5 SA sessions for GT, INT, and ST groups. A two-way ANOVA (PCA group × day) found no significant main effect of group (*F*(2,42) = 0.5, *p* = 0.59) or group × day interaction in cocaine intake (*F*(7.8,163.9) = 0.9, *p* = 0.56), indicating no difference between GT, INT, and ST groups across sessions. There was, however, a main effect of day (*F*(3.9,163.9) = 3.3, *p* < 0.05), indicating a significant change in cocaine intake across sessions. A paired samples *t* test suggested that cocaine intake significantly increased over time, as intake on the final FR5 session was significantly higher than that on the first FR5 session (all animals: *t*(44) = 4.8, *p* < 0.0005). Figure 2c shows the mean (± SEM) number of active and inactive nosepokes during non-drug periods across all FR5 SA sessions for GT, INT, and ST groups. A three-way ANOVA (PCA group × day × nosepoke) revealed a significant effect of nosepoke (*F*(1,42) = 33.0, *p* < 0.0005), but no other significant effects (group: *F*(2,42) = 0.5, *p* = 0.64; day: *F*(6.7,282.0) = 1.6, *p* = 0.13; group × day: *F*(13.4,282.0) = 0.8, *p* = 0.68; group × nosepoke: *F*(2,42) = 1.7, *p* = 0.20; day × nosepoke: *F*(7.7,324.9) = 1.5, *p* = 0.17; group × day × nosepoke: *F*(15.5,324.9) = 0.8, *p* = 0.70), indicating a distinction between the active and inactive nosepokes, but no other differences between the groups across sessions.

### 3-CRIT

**Fig. 3:**
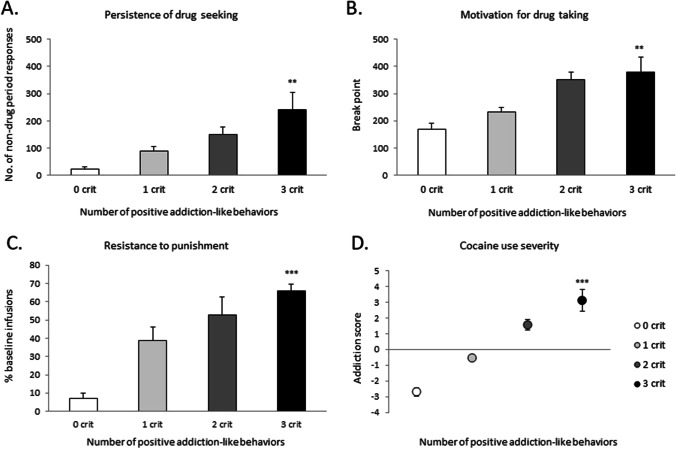
Addiction-like behavior in rats exhibiting 0, 1, 2, or 3 positive criteria (crit). As compared to 0 crit rats (*n* = 8), 3 crit rats (*n* = 5) displayed a higher score in every criterion for addiction-like behavior. **a** Persistence of cocaine-seeking, measured by the number of active nosepokes when cocaine is signaled as unavailable. **b** Motivation for cocaine-taking, measured as the breakpoint during PR. **c** Cocaine-taking and cocaine-seeking despite adverse consequences, measured by resistance to punishment. **d** Cocaine use severity, indicated by the addiction score. 0 and 3 crit animals differed significantly in all measurements. Data represent the mean (± SEM) of each criterion/addiction score. **p < 0.005, ***p < 0.0005, relative to 0 crit

Following cocaine SA training, animals were scored and divided into 4 groups corresponding to the number of positive criteria they met as follows: 0 crit (*n* = 8, 18%), 1 crit (*n* = 21, 47%), 2 crit (*n* = 11, 24%), and 3 crit (*n* = 5, 11%). Figure 3a–d shows descriptive statistics for each of the 3 criteria and addiction scores. Independent-samples *t* tests revealed that 0 and 3 crit animals differed significantly on all 4 measures: (a) persistence to drug-seeking as indicated by non-drug period responding (*t*(11) = 4.4, *p* < 0.005), (b) breakpoint in PR (*t*(11) = 4.2, *p* < 0.005), (c) resistance to punishment (*t*(11) = 11.9, *p* < 0.0005), and (d) addiction score (*t*(11) = 9.4, *p* < 0.0005). The prevalence of each PCA phenotype in the crit groups is as follows: 0 crit (3 GTs, 3 INTs, 2 STs), 1 crit (3 GTs, 10 INTs, 8 STs), 2 crit (2 GTs, 5 INTs, 4 STs), and 3 crit (0 GT, 2 INTs, 3 STs).

### PCA 3-CRIT score analyses

**Fig. 4:**
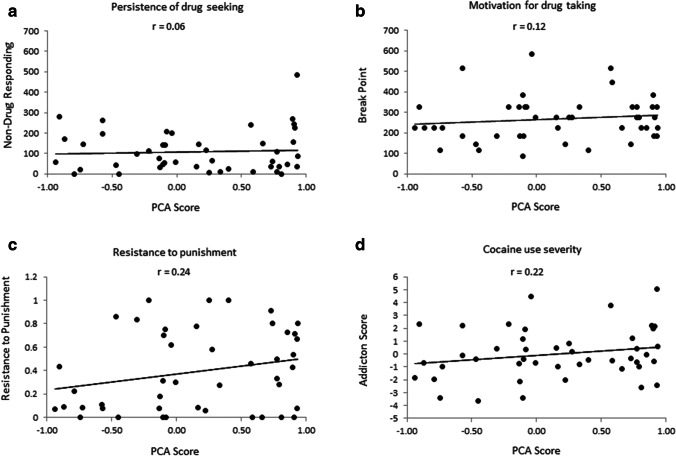
Correlation between PCA score and addiction criteria. There were no significant positive correlations between PCA score and **a** persistence of cocaine-seeking, **b** motivation for cocaine-taking, **c** resistance to punishment, or **d** addiction score. Data represent Pearson’s correlation coefficient (*r*) between pre-cocaine self-administration PCA scores and addiction criteria

There were no significant correlations between PCA score and addiction criteria as measured using the 3-CRIT model. Figure 4a–d shows Spearman’s correlation of PCA scores versus (a) persistence to drug-seeking as indicated by non-drug period responding (*r* = 0.06, *p* = 0.70), (b) breakpoint in PR (*r* = 0.12, *p* = 0.42), (c) resistance to punishment (*r* = 0.24, *p* = 0.11), and (d) addiction score (*r* = 0.22, *p* = 0.15). Thus, PCA score in the current experiment was not predictive of subsequent addictive-like behavior.

**Fig. 5:**
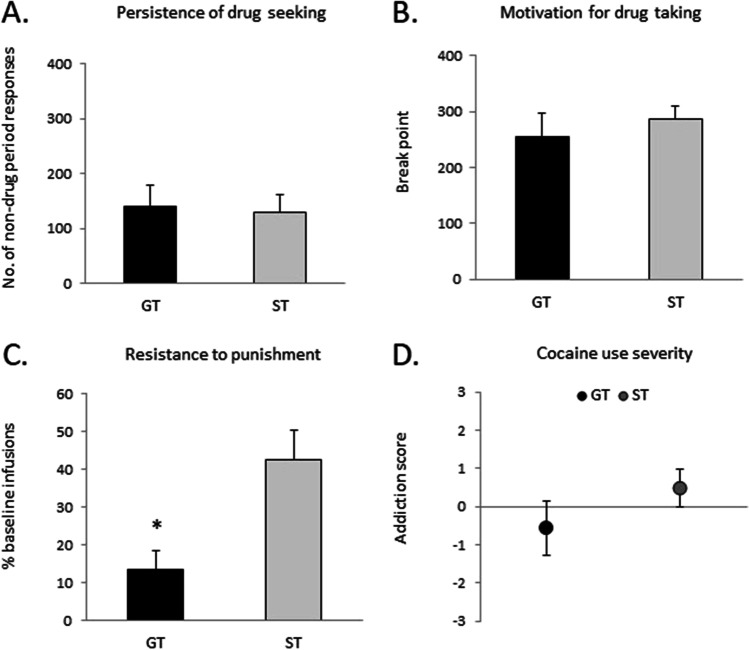
Addiction-like criteria in rats characterized as sign- and goal-trackers. There were no differences in **a** persistence of cocaine-seeking or **b** motivation for cocaine-taking between GTs and STs. Data represent the mean (± SEM) number of nosepokes during non-drug periods and breakpoint, respectively. **c** There was a significant difference between GTs and STs during the resistance to punishment session, with STs showing a higher resistance to punishment. Data represent the mean (± SEM) percentage of infusions relative to baseline. **d** There was no difference in addiction score between GTs and STs. Data represent mean (± SEM) addiction score as defined in the “Materials and methods” section. *p < 0.05

The 3-CRIT scores of GTs and STs were analyzed to determine whether the two extremes differed in addiction-like characteristics. There were no differences between GTs and STs on persistence of drug-seeking, PR breakpoint, or addiction score, but a significant difference in resistance to punishment. Figure 5a–d shows the mean (± SEM) non-drug period responding, PR breakpoint, resistance to punishment, and addiction scores, respectively, in GTs and STs. Independent-samples *t* tests revealed that GTs and STs did not differ on non-drug period responding (*t*(23) = 0.2, *p* = 0.83), PR breakpoint (*t*(23) = 0.7, *p* = 0.48), or addiction score (*t*(23) = 1.2, *p* = 0.25), but STs showed significantly greater resistance to punishment than GTs (*t*(23) = 2.4, *p* < 0.05).

## Discussion

The aim of the present study was to investigate whether prolonged daily cocaine self-administration resulted in a differential expression of addiction-like behaviors in GTs, INTs, and STs that would correlate with PCA scores defining these subgroups. Our results showed no correlations between PCA scores and either persistence of drug-seeking, motivation to self-administer cocaine, drug-taking despite adverse consequences, or overall addiction score. These findings are in line with previous work using a different study design and different indicators to assess addiction-like behavior (Kawa et al. 2016). Given that addiction is a chronic, oftentimes lifelong problem, these findings of no correlation between PCA score and addictive-like behaviors following prolonged drug experience are especially important because they suggest that PCA phenotyping along the continuum of GT, INT, and ST scores may not be a valid predictor of the vulnerability to addiction-like characteristics measured using the 3-CRIT model of cocaine addiction.

The PCA procedure used here showed that the majority of rats were scored as INTs, followed by STs and GTs. Similar results regarding this population distribution have been previously reported (Fitzpatrick and Morrow 2016). However, these findings are in contrast with previously reported findings from our lab that showed a higher percentage of GTs than STs in the population of animals (Enkel et al. 2019), although this reversed distribution has also been detected in our lab (unpublished finding of Thomas Enkel). Furthermore, Fitzpatrick et al. (2013) demonstrated a variation in PCA behaviors among Sprague Dawley rats even between animals from the same vendor but different colonies, which may have contributed to this altered distribution.

We obtained a lower percentage of 0 crit animals than previous 3-CRIT studies. However, Kasanetz et al. (2013) reported a similar population distribution. Importantly, the percentage of addicted-like individuals (3 crit) demonstrated here was similar to previously reported results from our lab (Cannella et al. 2013), as well as among other studies in which 3 crit animals represented the lowest percentage of the population (Belin et al. 2009; Kasanetz et al. 2013). Furthermore, cocaine intake was consistent among the subgroups of animals (0, 1, 2, and 3 crit), with no difference in the number of infusions achieved prior to 3-CRIT testing, which is also in accordance with previous findings (Belin et al. 2009; Cannella et al. 2013; Deroche-Gamonet et al. 2004).

Previous studies using limited drug exposure have suggested that STs may be more vulnerable to the development of addiction-like behavior than GTs, a premise primarily based on several studies that have been conducted in recent years exploring this hypothesis with respect to the incentive sensitization theory of addiction (Robinson and Berridge 1993, 2000). According to this theory, addiction develops into compulsive behavior when sensitization of the mesolimbic system to the incentive motivational properties of drug and drug-associated cues occurs, ultimately increasing the “wanting” of drug-associated cues (Robinson and Berridge 1993, 2000). Recent studies have shown that STs are initially more motivated to administer cocaine (Saunders and Robinson 2011) and choose cocaine over food as non-drug reward (Tunstall and Kearns 2015). In addition, STs are more impulsive (Flagel et al. 2010; Lovic et al. 2011), have a relatively weak degree of top-down attentional control (Enkel et al. 2019; Paolone et al. 2013; Sarter and Phillips 2018), and are more receptive to discrete cocaine cues compared to GTs (Saunders and Robinson 2010; Saunders et al. 2013; Yager and Robinson 2013). In contrast, GTs may be more vulnerable than STs to contextual cues or “occasion setters” (i.e., discriminative stimulus, DS), which are thought to utilize higher-order hierarchical control over behavior (Sarter and Phillips 2018) and are able to renew drug-seeking behavior in both rats (Crombag and Shaham 2002; Fuchs et al. 2005) and humans (Mayo et al. 2013; O’Brien 2005). GTs have also been demonstrated to show increased conditioned hyperactivity and renewal of cocaine-seeking induced by the original cocaine context following extinction in a novel context relative to STs (Saunders et al. 2014). These findings suggest distinct mechanisms and neural systems necessary for processing motivationally salient information, as well as the possibility of varying triggers and routes to substance dependence (Flagel and Robinson 2017; Robinson et al. 2014) that are relevant for the study of addiction but not measured in the current study.

Direct comparison of ST and GT behavior for each of the 3 addiction criteria or overall addiction score showed that these phenotypes did not differ in persistence of drug-seeking, motivation for drug-seeking, or addiction score; however, there was a significant difference found in resistance to punishment. Sign-trackers displayed significantly higher resistance to punishment compared to GTs, suggesting that among some attributes of addiction-like behavior, STs may, in fact, be more susceptible than GTs. These findings are in contrast to a previous report demonstrating that, although not in the context of prolonged cocaine experience, GTs and STs did not differ in cocaine SA when preceded by footshock punishment (Saunders et al. 2013). However, in the same study, the authors demonstrated that STs were more willing than GTs to nosepoke following the non-contingent presentation of a cue light previously associated with cocaine following an incubation period (Saunders et al. 2013). In addition, a few studies regarding PCA behavior and fear conditioning have been performed. For example, a study of acute fear conditioning reported a higher freezing response in the presence of discrete cues predicting footshock in STs, whereas GTs showed a greater contextual freezing response, despite the two groups demonstrating identical fear acquisition, suggesting no differences in pain sensitivity (Morrow et al. 2011). Morrow et al. (2015) also reported less freezing in STs relative to GTs 3 days after conditioning, but similar freezing levels between the two groups after 33 days, indicating a fear incubation effect present in STs but not GTs. A common thread regarding these and the resistance to punishment criterion in the current study appears to be the greater valence attribution by STs to discrete stimuli as discussed here and elsewhere (e.g., reviewed in Flagel and Robinson 2017; Sarter and Phillips 2018) that results in—in the case of fear—increases in conditioned freezing behavior and, in response to the presence of drug cues, an increase in conditioned motivational drug-seeking (Milton and Everitt 2010). What remain unclear are the precise circumstances in which these differential conditioned behaviors occur, and further studies are necessary to elucidate the processes governing the increased resistance to punishment in STs relative to GTs.

Substance use disorder is a chronic, complex, and relapsing disorder characterized by a variety of symptoms related to a loss of control over drug intake in humans. Despite the complexity of addiction and the difficulty in modeling it in all of its breadth in preclinical conditions, there are several preclinical models that focus on different features of addiction-like behavior. The 3-CRIT model of cocaine addiction attempts to model certain diagnostic criteria selected from DSM-IV, briefly the (1) inability to refrain from seeking the drug (persistence), (2) motivation for the drug (PR), and (3) continued use of the drug despite negative consequences (resistance to punishment) (American Psychiatric Association, DSM-IV 2000; Belin and Deroche-Gamonet 2012) following extended cocaine SA in rats. In addition to the psychological processes involved in addiction that may mediate differences in GTs and STs, as discussed above, other features of addiction not measured as part of the 3-CRIT model may also limit its ability to identify differences among these two subsets. Relapse is a critical feature of addiction, and the modeling of relapse behavior in animals using, for example, cue-induced reinstatement procedures, in which maladaptive cue-focused behavior can be more readily examined, may be a more valuable tool in identifying features of addiction that can be predicted by GT and ST behaviors. In addition to the incubation/reinstatement study mentioned above (Saunders et al. 2013), other studies have detected differences between GTs and STs in reinstatement studies (Everett et al. 2020; Pitchers et al. 2017). The 3-CRIT model, despite employing both DS and CS, does not extensively focus on the important role of occasion setters or conditioned cues in driving drug-seeking behaviors as part of the testing criteria, and thus, including an incubation or extinction and reinstatement protocol following testing may expose differences between GTs and STs. A systematic evaluation of the role of goal- and sign-tracking among 0 and 3 crit animals under relapse-like conditions has not been performed. It is also important to note that the 3-CRIT model does not capture other, equally important aspects of addiction, such as social context, tolerance, and withdrawal that also contribute to the complexity of drug dependence.

In conclusion, we report no significant correlations between PCA scores and addiction-like criteria following prolonged cocaine self-administration in the 3-CRIT model. The findings presented here suggest that behavior along the continuum of GT, INT, and ST PCA scores may not be a reliable predictor of the onset or severity of cocaine addiction with respect to those addiction-like characteristics examined using the 3-CRIT model. However, resistance to punishment may be one feature of the 3-CRIT model that may differ between GTs and STs and thus requires further evaluation. This study adds additional information to an increasing number of studies examining the role of goal- and sign-tracking phenotypes in addiction models.
