# Correction to: Sign‑ and goal‑tracking score does not correlate with addiction‑like behavior following prolonged cocaine self‑administration

**DOI:** 10.1007/s00213-021-05891-y

**Published:** 2021-06-16

**Authors:** Veronika Pohořalá, Thomas Enkel, Dusan Bartsch, Rainer Spanagel, Rick E. Bernardi

**Affiliations:** 1 Institute of Psychopharmacology, Central Institute of Mental Health, Medical Faculty Mannheim, University of Heidelberg, J5, 68159 Mannheim, Germany; 2 Department of Molecular Biology, Central Institute of Mental Health, Medical Faculty Mannheim, University of Heidelberg, Mannheim, Germany

**Correction to: Psychopharmacology**

10.1007/s00213-021-05858-z

The y-axis in panel C resistance to punishment in Figures 3 and 5 is mislabeled "% baseline AP" when it should read "% baseline infusions or just "% baseline."

The original article has been corrected.

